# Pre-reproductive maternal enrichment influences *rat* maternal care and offspring developmental trajectories: behavioral performances and neuroplasticity correlates

**DOI:** 10.3389/fnbeh.2015.00066

**Published:** 2015-03-12

**Authors:** Debora Cutuli, Paola Caporali, Francesca Gelfo, Francesco Angelucci, Daniela Laricchiuta, Francesca Foti, Paola De Bartolo, Elisa Bisicchia, Marco Molinari, Stefano Farioli Vecchioli, Laura Petrosini

**Affiliations:** ^1^Department of Psychology, University “Sapienza” of RomeRome, Italy; ^2^Santa Lucia FoundationRome, Italy; ^3^Department of Systemic Medicine, University of Rome Tor VergataRome, Italy; ^4^Department of Sociological and Psychopedagogical Studies, University “Guglielmo Marconi” of RomeRome, Italy; ^5^Institute of Cell Biology and Neurobiology, National Research CouncilRome, Italy

**Keywords:** environmental enrichment, BDNF, reelin, neurogenesis, cognition, maternal behavior

## Abstract

Environmental enrichment (EE) is a widely used paradigm for investigating the influence of complex stimulations on brain and behavior. Here we examined whether pre-reproductive exposure to EE of female rats may influence their maternal care and offspring cognitive performances. To this aim, from weaning to breeding age enriched females (EF) were reared in enriched environments. Females reared in standard conditions were used as controls. At 2.5 months of age all females were mated and reared in standard conditions with their offspring. Maternal care behaviors and nesting activity were assessed in lactating dams. Their male pups were also behaviorally evaluated at different post-natal days (pnd). Brain BDNF, reelin and adult hippocampal neurogenesis levels were measured as biochemical correlates of neuroplasticity. EF showed more complex maternal care than controls due to their higher levels of licking, crouching and nest building activities. Moreover, their offspring showed higher discriminative (maternal odor preference T-maze, pnd 10) and spatial (Morris Water Maze, pnd 45; Open Field with objects, pnd 55) performances, with no differences in social abilities (Sociability test, pnd 35), in comparison to controls. BDNF levels were increased in EF frontal cortex at pups' weaning and in their offspring hippocampus at pnd 21 and 55. No differences in offspring reelin and adult hippocampal neurogenesis levels were found. In conclusion, our study indicates that pre-reproductive maternal enrichment positively influences female rats' maternal care and cognitive development of their offspring, demonstrating thus a transgenerational transmission of EE benefits linked to enhanced BDNF-induced neuroplasticity.

## Introduction

Environmental enrichment (EE) is a widely used paradigm for investigating the influence of complex sensori-motor, cognitive and social stimulations on brain and behavior displayed during both development and adulthood in different experimental conditions (Rosenzweig et al., 1964; Nithianantharajah and Hannan, 2006). EE exerts beneficial effects on many behavioral (improved motor, cognitive and emotional performances, reduced stress reactivity), morphological (increase in brain weight, dendritic branching, number of spines, synaptic density, and neurogenesis), and molecular (changes in gene expression, modulation of neurotrophic and neurotransmitter systems) brain features (Nithianantharajah and Hannan, 2006, 2009; Petrosini et al., 2009; Baroncelli et al., 2010; Simpson and Kelly, 2011; Sale et al., 2014).

Despite the huge literature on the beneficial effects of EE applied immediately after weaning, in adulthood or even in aging, the transgenerational proactive effects of pre-reproductive EE have been only recently investigated (Arai et al., 2009; Arai and Feig, 2011; Leshem and Schulkin, 2012; Mashoodh et al., 2012; Caporali et al., 2014). The transfer of parents' phenotypic traits to the offspring is a debated process in biology since its promotion by Lamarck (Lamarck, 1809). This epigenetic (non-genetic) phenomenon of imprinting of parental environmental experiences on the offspring genome can lead to a different offspring phenotype that can persist over generations (Weaver, 2007).

The neurobehavioral consequences of the interactions between individual and environment take place as early as conception and continue to be important across the life. During embryonic and fetal development, activation of orchestrated chains of genes is the prime driving force directing central nervous system maturational processes. However, besides the overwhelming impact of the genetic control, even the environmental stimuli strongly influence the highly susceptible developing structures. Namely, the environment experienced by the pregnant mother exerts substantial effects on the intrauterine milieu and alters fetal organogenesis. During pregnancy, the maternal exposure to environmental toxins, pollutants, radiations, drugs, or alcohol, hormonal alterations (e.g., prenatal stress), food deprivation, or micronutrient deficiency have a deleterious impact on physical and behavioral offspring development (Rice and Barone, 2000; Meck and Williams, 2003; Van den Bergh et al., 2005; Weinstock, 2005; Mueller and Bale, 2008; Swanson et al., 2009; Thompson et al., 2009; Charil et al., 2010; Glover, 2011). On the other hand, even positive neurodevelopmental effects may occur, as in the case of the prenatal exposure to complex environments (McKim and Thompson, 1975; Kiyono et al., 1985; Dell and Rose, 1987; Welberg et al., 2006; Leshem and Schulkin, 2012; Mychasiuk et al., 2012; Rosenfeld and Weller, 2012). For example, voluntary exercise during pregnancy evokes positive and long-lasting effects on the offspring behavioral performances by increasing hippocampal neurogenesis and brain-derived neurotrophic factor (BDNF) expression (Parnpiansil et al., 2003; Bick-Sander et al., 2006; Lee et al., 2006; Sale et al., 2007; Herring et al., 2012).

Also after birth the newborn remains very sensitive to the (positive or negative) action of the environment. Given the primary source of environmental stimulation is represented by the mother, pup's post-natal development is mainly sculptured by maternal care (Francis and Meaney, 1999; Meaney, 2001; Macrì and Wurbel, 2006; Champagne and Meaney, 2007; Rosenfeld and Weller, 2012). Notably, maternal care may be in turn altered by the environmental factors experienced by the mothers. For example, stressed mothers display abusive behaviors on the pups that result in persisting changes in BDNF gene expression in the prefrontal cortex of the adult offspring (Roth et al., 2009). Also spontaneous variations in the quality or quantity of maternal care leave long-lasting epigenetic marks on offspring molecular factors crucial for plasticity, such as expression of BDNF, NMDA and glucocorticoid receptors (Weaver et al., 2004; Champagne and Meaney, 2006; Weaver, 2007; Champagne and Curley, 2009). Nevertheless, the relationship of prenatal exposure to EE, maternal care and offspring cognitive development has never been investigated. In fact, the few studies addressing the pre-reproductive and prenatal EE effects on offspring behavior neglect the issue of maternal care (Arai et al., 2009; Mychasiuk et al., 2012). One study addressing the EE exposure effects during pregnancy reports enhanced licking levels, but does not provide information on cognitive development of the offspring (Sale et al., 2004). Another study reports that early pre-reproductive EE transgenerationally influences offspring motor and cognitive behavior, but does not provide information on maternal care (Leshem and Schulkin, 2012; Caporali et al., 2014). Finally, modifications of maternal care are reported in standard reared females mated with enriched male, but no information on offspring cognitive abilities are provided (Mashoodh et al., 2012).

It is well known that neurotrophins, as the BDNF, influence brain development, neuronal plasticity (synapse formation, axonal outgrowth, and circuital remodeling), as well as plastic mechanisms involved in learning and memory, and in the response to stress or injury (Pham et al., 2002; Segal, 2003; Blum and Konnerth, 2005; Branchi et al., 2006; Angelucci et al., 2009; Gelfo et al., 2011). The expression of offspring neurotrophins is modified by early experiences, either negative (i.e., maternal maltreatment) or positive (i.e., environmental enrichment) (Sale et al., 2004; Roth et al., 2009; Caporali et al., 2014). Interestingly, early enrichment affects the BDNF epigenetic structure without altering its expression (Branchi et al., 2011). Moreover, it has been shown that the offspring of mothers that exhibit high levels of pup licking have an increased BDNF expression in the hippocampus (Liu et al., 2000). A recent study by our team (Caporali et al., 2014) has shown that the offspring of enriched mothers display anticipated development of complex motor behaviors associated with increased BDNF and nerve growth factor (NGF) levels in the principal motor areas, striatum and cerebellum.

On such a basis the present study was aimed at analyzing whether and how pre-reproductive exposure to EE of female rats may influence their maternal care as well as cognitive performances of their offspring. To this aim, from weaning to breeding age, the enriched female rats were reared in large groups in a highly stimulating environment with different objects frequently changed. Females used as controls were pair-housed in standard conditions. At 2.5 months of age all females were mated and reared in standard conditions with their offspring, which thus never experienced the environmental enrichment. Maternal care behaviors and nesting activity were assessed in lactating dams. At different post-natal days (pnd), their male pups were behaviorally evaluated for discriminative, social and cognitive abilities. Given adult hippocampal neurogenesis is a form of structural and functional plasticity particularly sensitive to environmental modifications (Masiulis et al., 2011; Kempermann, 2012), at the end of behavioral testing it was measured in the dentate gyrus of adult pups. Furthermore, to gain information on the functional modifications induced by pre-reproductive maternal EE exposure, BDNF expression in frontal cortex, hippocampus, and cerebellum was evaluated in dams at pups' weaning and in the offspring at birth, weaning and adulthood. Finally, due to its integral role in brain development and adult neurogenesis (Masiulis et al., 2011), hippocampal and cortical levels of the structural protein reelin were also measured in the pups at birth and in adulthood.

## Materials and methods

### Experimental design

#### Maternal housing conditions

At weaning (pnd 21) 20 female Wistar rats (54.73 ± 1.99 g) were randomly assigned to enriched or standard rearing conditions.

From pnd 21 to 72 Enriched Females (EF) were housed in group of 10 in a large cage (100 × 50 × 80 cm) with an extra level constructed of galvanized wire mesh and connected by ramps to create two interconnected levels, according to the enrichment conditions previously described (Petrosini et al., 2009; Cutuli et al., 2011; Foti et al., 2011; Caporali et al., 2014). The cage contained wood sawdust, two running wheels, a shelter (a house-shaped toy with a concave opening in which the rat could enter), colored plastic toys (red or green small balls, little bells, jingle noise-maker playthings, and ropes), and small objects (transparent rat igloo, colored bricks, cubes, tunnels, a mirror, and platform). Throughout the enrichment period, the shelter and running wheels were kept in the cage and the toys and constructions were changed twice a week. Once a week, the feeding boxes and water bottles were moved to different cage areas to promote explorative behaviors. Furthermore, each enriched animal was handled daily for at least 10 min.

Standard Females (SF) were pair-housed in cages (40 × 26 × 18 cm) containing wood sawdust, a red plastic tube and no toys. Food and water were delivered *ad libitum* through dispensers kept always in the same position. SF received the usual care by the animal house staff without any extra-manipulations. This procedure avoided an impoverished rearing and allowed being accustomed to the human contact.

A 12/12 h dark/light cycle (light on between 07:00 and 19:00 h) was applied to both EF and SF groups. On the pnd 72, all females were weighted, and EF were pair-housed in standard cages. After a week, each EF and SF female in oestrus stage was caged for 5 days with a standard-reared male rat (≈300 g) to allow mating. Afterwards, the males were removed, and the females were maintained in the standard home cages throughout pregnancy, delivery and until offspring's weaning.

All efforts were made to minimize animal suffering and reduce the number of animals that were used, per the European Directive (2010/63/EU). All procedures were approved by the Italian Ministry of Health.

#### Experimental groups of pups

At birth (pnd 0), litter size and sex ratio were noted. Then, culling of the litter was quickly performed (all procedures taking max 5 min) reducing the litters to six males and two females. Litters not compliant with this condition were excluded from biochemical and behavioral analyses. Thus, litters from six EF and SF dams were used. Depending on the maternal rearing conditions, two groups of male pups were obtained: one group encompassed the pups of EF (group name: EF-p; *n* = 24); the other group encompassed the pups of SF (group name: SF-p; *n* = 24). Note that the difference in rearing conditions concerned the mothers in their pre-reproductive phase and not the pups that were all reared in standard conditions.

Two pups/litter (*n* = 12/group) were behaviorally evaluated for discriminative (maternal odor preference T-maze test on pnd 10), social (Sociability test on pnd 35) and cognitive abilities (Morris Water Maze, MWM, on pnd 45, and Open Field with objects, OF, on pnd 55). A sub-group of pups randomly selected among the behaviorally tested ones was used to perform BrdU analyses at the end of behavioral procedures (pnd 55; *n* = 6/group).

Other two pups/litter (*n* = 12/group) not previously behaviorally tested were submitted to neurochemical analyses to measure levels of BDNF (at three time-points: pnd 0, 21, 55; *n* = 4/group/time-point) and reelin (at two time-points: pnd 0, 55; *n* = 4/group/time-point).

BDNF determination was also performed on dams (*n* = 4/group) at *post-partum* day (ppd) 21.

**Figure 1:**
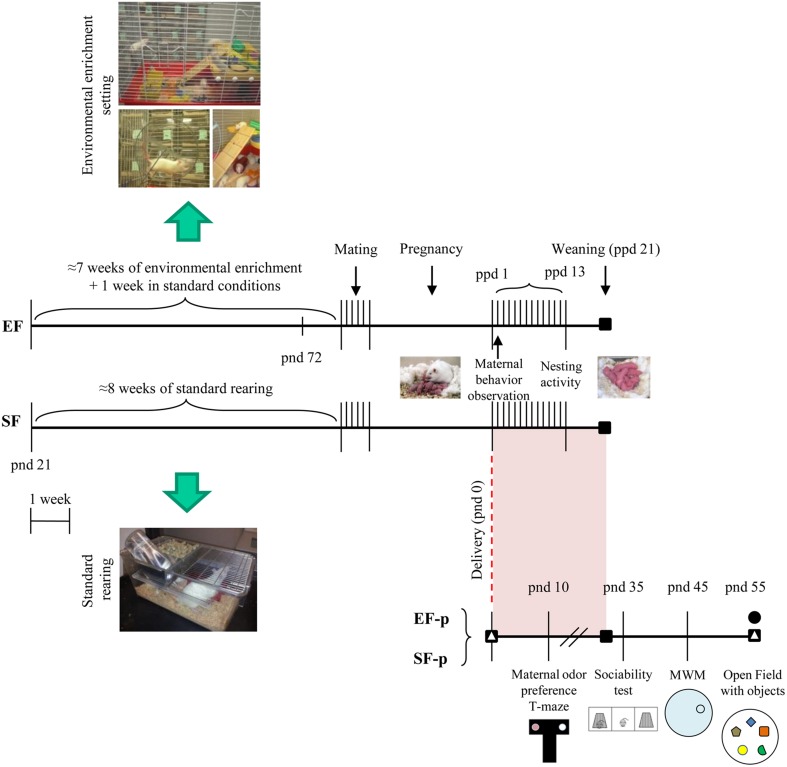
**Timeline of the experimental procedure**. Experimental groups of female rats according to different pre-reproductive rearing conditions (EF, Enriched Females; SF, Standard Females. Duration: ≈7 weeks). Experimental groups of male pups (EF-p, Enriched Females' pups; SF-p, Standard Females' pups), behavioral testing (maternal odor preference; Sociability test; Morris Water Maze, MWM; Open Field with objects, OF) and biochemical analyses (■, neurotrophin determination; Δ, reelin quantification; •, hippocampal neurogenesis analyses).

Pup weight was collected at pnd 10, 35, and 55. Siblings of the behaviorally or neurochemically analyzed pups were devoted to different researches. Global timing of experimental procedures is reported in Figure 1.

### Behavioral testing

#### Nest building activity

To test the nest-building ability and proneness, each day from the day of delivery (ppd 0) till ppd 13 dams were given the opportunity to build a nest providing them 6 g of sterile surgical cotton placed on the lid of each cage, as described in previous studies (Schneider and Lynch, 1984; Laviola et al., 1990; Petruzzi et al., 1995; Venerosi et al., 2008). Quantitative index of nest building activity was the *weight* of the residual cotton. To analyze nest building propensity, the built nests were removed on ppd 1, 2, 4, 6, 9, 12. After nest removal new cotton was provided and *latency* to manipulate it was assessed. Nest building started when the female pulled down the cotton from the cage lid with forelimbs and mouth. The cut-off time was 10 min.

The *nest quality* was assessed daily by two independent observers blind to the pre-reproductive rearing condition of the dams (inter-rate reliability >0.9), using the following 4-point scale, modified from Kalueff et al. (2006) and Venerosi et al. (2008) (Supplementary Figure 1):

1. No nest (the dam did not build the nest or scattered the cotton and sawdust throughout the home cage with no clear nest shape);
2. Primitive flat nest (the dam used the cotton to build a plane nest);
3. Complex cup-shaped nest (the dam used the cotton to build an open nest with walls);
4. Complex hooded nest (the dam built a round and well-shaped nest with walls forming a ceiling). We recorded further qualitative nest features, as *position* (under or opposite the water bottle), *height* (lower, at the same level or higher than the middle height of the cage), *texture* (low, intermediate, high work out of the nest surface), and presence of *additional structures* (little agglomerates of cotton, sawdust or excrements put on the top of the cage).

#### Maternal behavior observations

Maternal behavior in rats consists of several behaviors toward the litter that ensure the pups' survival and promote offspring development. To obtain information about the effects of pre-reproductive environmental enrichment exposure of female rats on their maternal care at early stages, mother-pups interactions were recorded on ppd 1 (modified from Calamandrei and Keverne, 1994; Venerosi et al., 2008). All 30 min-observation sessions were made under dim light in a testing room out of the animal facility. The observations were made between 10.00 and 16.00 h and performed by a trained observer blind to pre-reproductive rearing condition of the dam. Animals were habituated to the testing room for 10 min. Thirty minutes before the start of each observation session, the pups were removed (isolation period), the nest eliminated, and the dam remained alone in her home cage. During the isolation period, the pups were weighted and then maintained altogether in a small box at 32° ± 1°C. This procedure allows to study maternal behavior in a condition eliciting it, not in basal level condition.

At the beginning of each observation, the cage lid was gently replaced by a transparent perforated Plexiglas top, the pups were re-placed in their home cage in the side opposite to the previously removed nest, and dam's behavior was video-recorded for 30 min. Latency, frequency and duration of the following behaviors (Fleming and Rosenblatt, 1974; Petruzzi et al., 1995; Venerosi et al., 2008) were collected:

- Pup-directed Behaviors:
  - *Retrieving*: the dam was picking up any pup in her mouth and carrying it to the nest;
  - *Licking*: the dam was licking or grooming any part of the pup's body, primarily the ano-genital region;
  - *Sniffing*: the dam was sniffing one or more pups;
  - *Nursing*: part of the litter was attached to dam's nipples while the dam did not show obvious back-arching;
  - *Crouching* (or arched-back nursing): the dam was domed over all pups with the body arched, hind-limbs splayed and no apparent movement;
  - *Nest Building*: the dam was pushing and pulling the sawdust toward the pups to form a nest.
- Non pup-directed behaviors:
  - *Digging*: the dam was nuzzling in the sawdust out of nest area, pushing and kicking it around using the snout and/or both fore- and hind-paws;
  - *Grooming*: the dam was wiping, licking, combing or scratching any part of its own body;
  - *Wall Rearing*: the dam was rearing on hindlimbs, while leaning (or not) with the forelimbs on the cage walls, often sniffing the air;
  - *Exploring*: the dam was moving around the cage and sniffing the substrate, but not carrying pups or nesting material;
  - *Resting*: the dam was lying down alone, out of the nest.
- *Other behaviors:* all behaviors different from the ones classified in the previous categories.

Manual scoring was performed by a researcher blind to pre-reproductive rearing condition of the dams by using Ethovision XT (Noldus, The Netherlands). Data analysis was performed on three 10 min-blocks.

#### Maternal odor preference T-maze test

To assess early discriminative performances and maternal preference behavior, pups were tested in an odor discrimination test at pnd 10 (eyelids still closed). The apparatus consisted in a black Plexiglas T-shaped maze (start arm: 25 × 9 cm; choice arms: 12.5 × 9 cm; height: 8.5 cm). Each pup was gently placed in the start arm and allowed to freely explore the maze for 5 min. Two glass cups (diameter: 2.5 cm; height: 2 cm) containing sawdust of the home cage with mother's excrements (maternal olfactory stimulus) or clean sawdust (neutral olfactory stimulus) were placed at the end of each choice arms. *Time* spent in the two arms and time spent in *direct contact* with the olfactory stimuli were recorded. After each testing session the apparatus was cleaned and the position of the olfactory stimulus was alternated among pups.

#### Sociability test

To quantitate sociability tendencies in rat adolescent pups we used the Sociability test (Nadler et al., 2004; Cutuli et al., 2013). The apparatus consisted of a rectangular three-chamber wooden box (150 × 40 × 40 cm) with black floor and white lateral walls. The central chamber was 30 cm-long and the two lateral chambers were 60 cm-long. The three chambers were divided by two transparent Plexiglas walls with an opened middle door (height: 10 cm; width: 8 cm), which allowed free access to each lateral chamber. Each lateral chamber contained a small plastic cage (diameter: 18 cm) with mesh-like holes in which a stranger rat was confined during the Sociability test. The experimental procedure consisted of two sessions, Habituation and Sociability test, with an inter-session interval of 3 min (Cutuli et al., 2013). During Habituation each rat was allowed to freely move for 10 min in the entire apparatus with the small cages maintained empty. During Sociability test an unfamiliar conspecific male rat of the same age (about 35–38 pnd) was placed inside one of the two small wire cages in the lateral chambers (randomly selected and counterbalanced for each group). The to-be-tested rat was placed in the central chamber and it was allowed to freely explore for 10 min the entire apparatus and contact the small wire cages. Rat behavior was recorded by a video camera mounted on the ceiling. The resulting video signal was relayed to a monitor and to an image analyzer (Ethovision XT, Noldus, The Netherlands).

The parameters analyzed were: *total distance* traveled, and *time* (s) spent in the three compartments; *frequency of entries* in the lateral compartments. In addition, time spent in a 9 cm-square (target) around the small wire cages (containing the social stimulus or empty) was calculated as *direct contact*.

#### Morris water maze

To assess spatial and procedural abilities, late adolescent rats were submitted to MWM (Cutuli et al., 2009). The rats were placed in a circular white pool (diameter 140 cm) filled with 24°C water made opaque by the addition of atoxic acrylic color (Giotto, Italy). An escape platform (diameter 10 cm) was submerged 2 cm below the water level in the NE quadrant. Each rat was submitted to a 10-trial Place phase followed by an 1-trial Probe phase with an inter-phase interval of 3 min. During Place trials, the rat was released into the water from randomly varied starting points and allowed to find the hidden platform for a maximum of 60 s with an inter-trial interval of 30 s. When the rat reached the platform, it was allowed to remain there for 30 s. If the rat failed to reach the hidden platform within 60 s, it was gently guided there by the experimenter. During Probe trial, the platform was removed and rat was allowed to swim for 30 s in searching for it. Navigational trajectories were recorded by a video camera whose signal was relayed to a monitor and to the previously described image analyzer.

The following MWM parameters were considered: *latencies* to find the platform; *total* and *peripheral distances* swum in the pool; mean *swimming velocity*; percentage of *time* spent in the previous rewarded quadrant (presence of platform) during Probe phase; *navigational strategies* put into action in reaching the platform. The navigational strategies were classified in four main categories (Cutuli et al., 2009), regardless the platform was reached or not: *Circling* (C), swimming in a 20-cm peripheral annulus, with inversion of swimming direction and counterclockwise and clockwise turnings in the peripheral sector of the pool; *Extended Searching* (ES), swimming around the pool in all quadrants, visiting the same areas more than once; *Restricted Searching* (RS), swimming not more than two pool quadrants, not visiting some tank areas at all; direct *Finding* (F), swimming toward the platform without any foraging around the pool. Two researchers who were unaware of the individual specimen's group categorized the swimming trajectories drawn by the image analyzer. They attributed the dominant behavior in each trial to a specific category. Categorization was considered reliable only when their judgments were consistent.

#### Open field with objects

To assess spatial discrimination and novelty recognition abilities, young adult rats were submitted to the OF test (Cutuli et al., 2009, 2013). The apparatus consisted of a circular arena (diameter 140 cm) delimited by a 30-cm-high wall. During session 1 (S1), each rat was allowed to freely move in the empty open field and its baseline activity level was measured. During the habituation phase (S2–S4), four objects were placed in a square arrangement in the middle annulus of the arena and a fifth one was placed in the central area. The five objects were: (1) a metal bar with a conical base; (2) a plunger; (3) a long steel rod; (4) a yellow rubber plug; and (5) a black cylinder with a plastic cup turned upside down on top of it. During the spatial change (S5 and S6), the spatial configuration was changed by moving objects 2 and 5 so that the initial square arrangement was modified to a polygon-shaped configuration, without any central object. During the novelty session (S7), the configuration was modified by substituting object 4 with a green plastic object shaped like a half moon. All sessions lasted 6 min with inter-session intervals of 3 min (Cutuli et al., 2009, 2013). Rats' behavior was recorded by a video camera whose signal was relayed to a monitor and to the previously described image analyzer.

In S1 the following motor and emotional parameters were analyzed: *total distance* (in cm) traveled in the arena; *peripheral distance*, percentage of the total distance traveled in a 20-cm peripheral annulus; number of *rearings*; number of *central crossings*; *motionless time*; *number of defecation boluses*. In S2–S4, the total *contact time* spent in contacting objects was analyzed. Contact was considered to have taken place when the rat's snout actually touched an object or when it sniffed the object for at least 1 s, but not when the rat leaned against, stood, or sat on the object. In S5–S7, the time spent contacting objects was expressed as *discrimination index* (d index), that is time exploring displaced (or novel) objects/total exploration time.

### Biochemical assays

#### Adult neurogenesis analyses

To correlate hippocampus-dependent behavioral performances (namely, in MWM and OF) with neuroanatomical changes in the hippocampal neurogenenic niche, the subgranular zone of the dentate gyrus, the hippocampal adult neurogenesis was examined. Twelve behaviorally tested animals (3 rats/litter/group) received three i.p. injections of 50 mg/kg BrdU dissolved in saline (0.9% NaCl adjusted to pH 7.2 with NaOH) during 1 day (inter-injection interval: 2 h). One day after the final injection the animals were sacrificed and transcardially perfused with 4% paraformaldehyde (PFA) in 0.1 M phosphate buffer (PBS), under deep anesthesia with chloral hydrate. The brains were removed and kept overnight in 4% PFA. Afterwards, brains were equilibrated in 30% sucrose and cryopreserved at −80°C.

The hippocampus from brains embedded in Tissue-Tek OCT (Sakura, USA) was cut by cryostat at −25°C in 40 μm coronal serial free-floating sections. To perform BrdU detection, DNA was denaturated with 2N HCl for 40 min at 37°C to facilitate antibody access, followed by 0.1 M borate buffer pH 8.5 for 20 min. Sections were incubated overnight at 4°C with a primary antibody rat anti-BrdU (AbD Serotec Cat# MCA2060 RRID:AB_323427) diluted 1:300 in TBS containing 0.1% Triton, 0.1% Tween 20 and 3% normal donkey serum (blocking solution). For immunofluorescence analysis, sections were stained for multiple labeling by using fluorescent methods. After permeabilization with 0.3% Triton X-100 in PBS, the sections were incubated with 3% normal donkey serum in PBS for 16–18 h with the following primary antibodies: 1:200 goat polyclonal antibodies against Doublecortin (DCX) (Santa Cruz Biotechnology, Inc. Cat# sc-8066). Secondary antibodies used to visualize the antigen were 1:200 donkey anti-rat Cy3-conjugated (Jackson ImmunoResearch; BrdU) 1:100, donkey anti-goat Cy2-conjugated (Jackson ImmunoResearch; Dcx). Images of the immunostained sections were obtained by laser scanning confocal microscopy by using a TCS SP5 microscope (Leica Microsystem; Germany).

#### Quantification of cell numbers

Quantitative analysis of hippocampal cell populations was performed by means of design-based (assumption-free, unbiased) stereology as previously described (Cutuli et al., 2014; Farioli-Vecchioli et al., 2014).

#### BDNF and reelin tissue processing

For BDNF and reelin determination, 4 animals were selected from each group of dams (EF and SF, at ppd 21) and not behaviorally tested pups (EF-p and SF-p, at pnd 0, 21, 55; 2 pups/dam). The animals were decapitated; the brains were quickly removed and dissected on ice by using a binocular dissection microscope. The following brain regions were collected according to method Glowinski and Iversen's, 1966: cortex and hippocampus (pups at pnd 0); frontal cortex, hippocampus and cerebellum (dams; pups at pnd 21, 55). Cerebellar neurotrophin expression was not analyzed at pnd 0, because of the very weak BDNF expression in this extremely immature area in the very early postnatal days, as previously described (Katoh-Semba et al., 1997; Sherrard and Bower, 2002). Brain regions were extracted in 1 ml extraction buffer/100 mg tissue. Brain tissue samples were homogenized in an ice-cold lysis buffer containing 137 mM NaCl, 20 mM Tris–HCl (pH 8.0), 1% NP40, 10% glycerol, 1 mM phenylmethanesulfonylfluoride (PMSF) 10 mg/ml aprotinin, 1 mg/ml leupetin, and 0.5 mM sodium vanadate. The tissue homogenate solutions were centrifuged at 14000 g for 25 min at 4°C. The supernatants were collected and stored at −80°C until analyses.

#### BDNF determination by enzyme-linked immunosorbent assay (ELISA)

As an index of the functional modifications induced by pre-reproductive maternal EE exposure, we evaluated in several brain regions the expression of BDNF, a neurotrophin specifically involved in synaptic plasticity changes supporting mnesic functions. BDNF concentrations were assessed using a two-site enzyme immunoassay kit (Promega, Madison, WI, USA). In brief, 96-well immunoplates (NUNC) were coated with 50 μl/well with the corresponding capture antibody which binds the neurotrophin, and stored overnight at 4°C. The next day serial dilutions of known amounts of BDNF ranging from 0 to 500 pg/ml were performed in duplicate to generate a standard curve. Then, the plates were washed three times with wash buffer and the standard curves and supernatants of brain tissue homogenates were incubated in the coated wells (100 μl each) for 2 h at room temperature (RT) with shaking. After additional washes, the antigen was incubated with second specific antibody for 2 h at RT, as specified in the protocol. The plates were washed again with wash buffer and then incubated with an anti-IgY HRP for 1 h at RT. After another wash, the plates were incubated with a TMB/Peroxidase substrate solution for 15 min and phosphoric acid 1M (100 μl/well) was added to the wells. The colorimetric reaction product was measured at 450 nm using a microplate reader (Dynatech MR 5000, Germany). BDNF concentrations were determined from the regression line for the BDNF standard (ranging from 7.8 to 500 pg/ml-purified mouse BDNF) incubated under similar conditions in each assay. Cross-reactivity with other related neurotrophic factors, for example, NT-3 and NT-4 was less than 3%. BDNF concentration was expressed as pg/g wet weight and all assays were performed in triplicate.

#### Reelin determination by western blot analyses

As further correlate of neuroplasticity reelin levels were assessed in both experimental pups groups. Protein content was quantified by Bradford's colorimetric assay (Bio-Rad, Milan, Italy). Each protein sample was separated by SDS-polyacrylamide gel electrophoresis and transferred to a nitrocellulose membrane. Membranes were saturated with 5% dried non-fat milk and incubated overnight with specific primary antibodies, such as mouse anti-Reelin (1:1000; Millipore, USA) and mouse anti-β-actin (1:5000; Sigma) (Biamonte et al., 2014). Membranes were then incubated with the appropriate horseradish peroxidase-conjugated secondary antibodies. Immunoreactive bands were detected by using an enhanced chemiluminescence kit (ECL; Amersham Biosciences).

### Statistical analysis

Statistical analyses were performed by using STATISTICA 8.0 (StatSoft). Data were firstly tested for normality (Wilk-Shapiro's test) and homoscedasticity (Levene's test). Then, behavioral data were analyzed by one-way and Two-Way ANOVAs for independent (group) and repeated measures (compartment, target, trial, strategy, session, object). Data on BDNF levels were analyzed by One-Way ANOVAs followed by Tukey's HSD test, separately for dams and pups. Reelin levels were analyzed using Student's *T*-test. When parametric assumptions were not fully met, data transformations (angular transformation for percentages) or non-parametric analyses of variance (Mann-Whitney's U, Friedman's test, Wilcoxon's test) were used. Differences were considered significant at the *p* = 0.05 level.

## Results

### Weight, litter size and sex ratio

At end of the EE exposure (pnd 72), EF (212.30 ± 6.90 g) weighted significantly less than SF (231.52 ± 3.23 g) [One-Way ANOVA: *F*_(1, 18)_ = 7.16, *p* = 0.01]. Pre-reproductive maternal enrichment did not affect litter size [EF: 11.0 ± 0.5 pups; SF: 11.6 ± 0.6 pups; One-Way ANOVA: *F*_(1, 18)_ = 0.54, *p* = 0.47] and male/female *ratio* [percentage of male pups: EF: 60.70 ± 2.17%; SF: 59.77 ± 4.12%; One-Way ANOVA: *F*_(1, 18)_ = 0.04, *p* = 0.84]. At pnd 1 EF male (6.64 ± 0.07 g) and female (6.61 ± 0.05 g) pups' weight was lower than SF male (7.25 ± 0.08 g) and female (7.05 ± 0.09 g) pups' one [One-Way ANOVA: male pups, *F*_(1, 70)_ = 12.02, *p* = 0.0009; female pups, *F*_(1, 22)_ = 7.59, *p* = 0.01]. No differences were found in the weight of male pups that underwent the behavioral testing during the successive time-points [pnd 10: EF-p: 22.80 ± 0.74 g; SF-p: 23.80 ± 1.08 g; One-Way ANOVA: *F*_(1, 22)_ = 0.64, *p* = 0.43; pnd 35: EF-p: 134.72 ± 2.19 g; SF-p: 130.36 ± 3.12 g; One-Way ANOVA: *F*_(1, 22)_ = 1.30, *p* = 0.27; pnd 55: EF-p: 205.61 ± 4.92 g; SF-p: 210.89 ± 3.45 g; One-Way ANOVA: *F*_(1, 22)_ = 0.77, *p* = 0.39].

### Behavioral testing

#### Dams

#### Nest building activity

**Figure 2:**
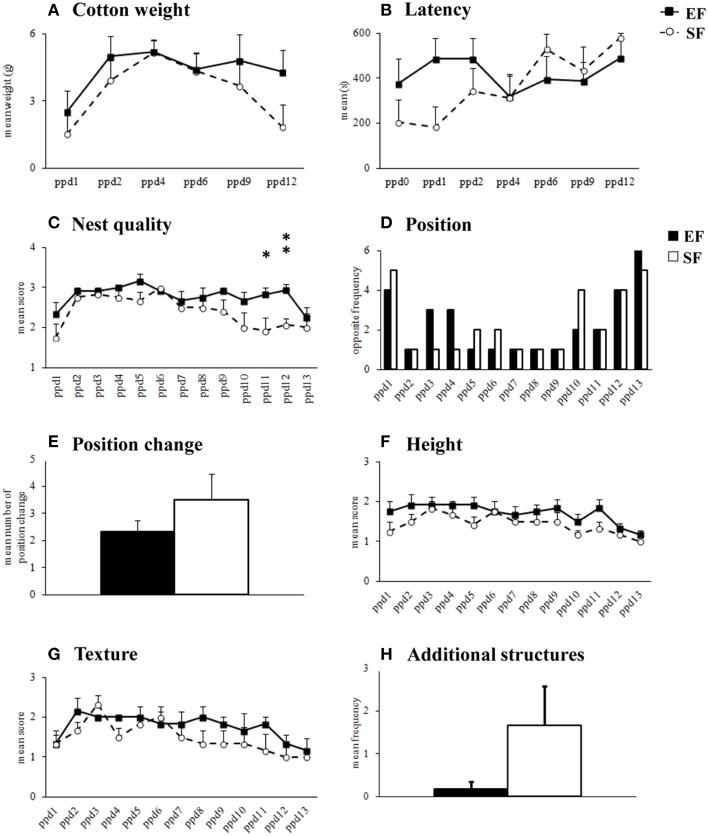
**Nest building activity**. Results of pre-reproductive maternal rearing condition on nest building activity are depicted. Quantitative (cotton weight, **A**; latency, **B**) and qualitative indexes (nest quality, **C**; position, **D**; position change, **E**; height, **F**; texture, **G**; additional structures, **H**) of nest building activity analyzed in EF and SF from the day of delivery (ppd 0) to ppd 13 (^*^p < 0.05; ^**^p < 0.01). Results are reported as mean ± SEM.

Non parametric analyses (Mann-Whitney's U) performed on *cotton weight* used by dams in their nest building activity (Supplementary Table 1) as well as on the *latency* to manipulate cotton (Supplementary Table 1) revealed that the EF and SF did not significantly differ (Figures 2A,B). Friedman's test performed on cotton weight used during the six days of observation revealed a similar inverted U-shaped trend in both groups (EF, *p* = 0.04; SF, *p* = 0.02). Namely, both EF and SF groups used more cotton during the intermediate days of observation (ppd 2–9) than in the first (ppd 1) and last (ppd 12) days. Friedman's test performed on latency to manipulate cotton revealed a significant trend to increase the latencies as observation days went only in SF (*p* = 0.004), while EF showed latencies not significantly different among days (*p* = 0.78).

As for *nest quality* score, EF obtained higher scores than SF in the final observation days (Supplementary Table 1) because still built complex cup-shaped nests on ppd 11 (*p* = 0.03) and 12 (*p* = 0.007), instead of primitive flat nest, as SF did (Figure 2C). Friedman's test revealed that in both EF and SF groups nest quality scores increased from ppd 1 onwards and decreased during the last observation days (EF, *p* = 0.0008; SF, *p* = 0.002).

No effect of pre-reproductive rearing condition was evident on the further qualitative nest features (Supplementary Table 1), as *position* (Figures 2D,E), *height* (Figure 2F), *texture* (Figure 2G), *additional structures* (Figure 2H).

In summary, in the 2nd post-natal week EF dams appeared to be longer engaged in building nests of higher shape complexity than SF.

#### Maternal behavior observations

**Figure 3:**
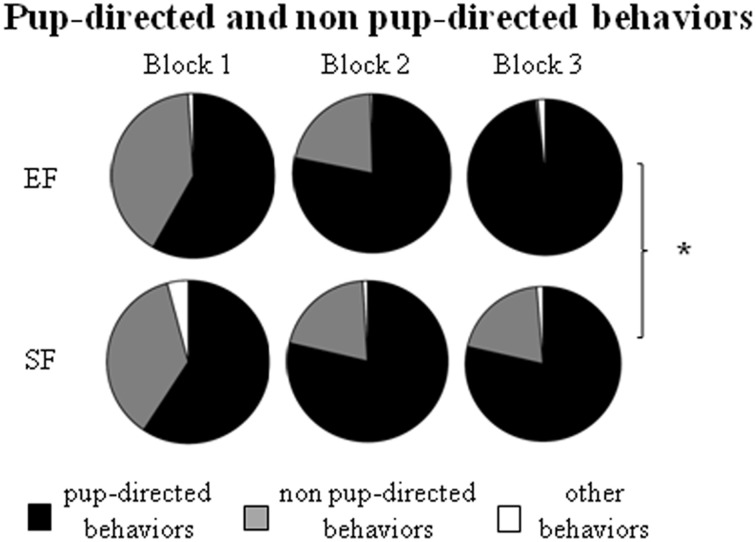
**Maternal behavior observation**. Results of pre-reproductive maternal rearing condition on maternal behavior are depicted. Sum of duration of the different kind of maternal behaviors (pup-directed, non pup-directed and other behaviors) in each of the three 10 min-blocks of observation. (^*^p < 0.05). Results are reported as mean ± SEM.

**Figure 4:**
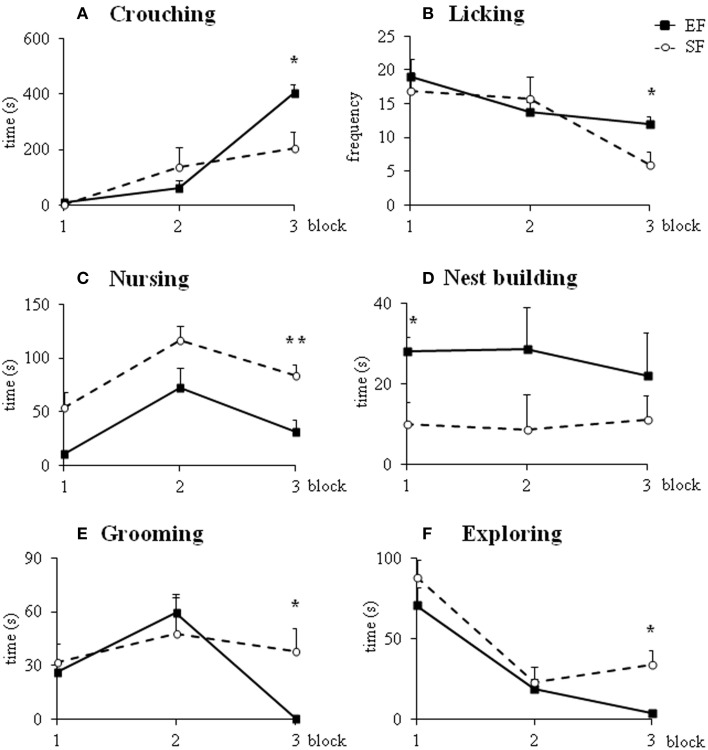
**Maternal behaviors**. Results of pre-reproductive maternal rearing condition on specific maternal behaviors are depicted. Line graphs represent duration and frequency of single pup-directed **(A–D)** and non pup-directed **(E–F)** maternal behaviors analyzed in EF and SF on ppd 1 (^*^p < 0.05; ^**^p < 0.01). Results are reported as mean ± SEM.

Non parametric analyses (Mann-Whitney's U) performed on the sum of *pup-directed behaviors* at ppd 1 revealed significant differences between EF and SF groups in the final phase (3rd 10 min-block) of the 30 min-observations, being EF longer engaged in pup-directed behaviors than SF (Figure 3; Supplementary Table 2A). Detailed analyses performed on single pup-directed behaviors demonstrated that during just the 3rd block of observation the EF showed higher *Crouching* duration (Figure 4A) and *Licking* frequency (Figure 4B), and lower *Nursing* duration and frequency than SF (Figure 4C; Supplementary Table 2A). Also during the 1st block of observation EF showed higher duration and frequency of *Nest Building* (Figure 4D; Supplementary Table 2A). Symmetrically, EF emitted less *non pup-directed behaviors* than SF during the 3rd block of observation (Supplementary Table 2B). Namely, EF engaged shorter and less frequent *Grooming* (Figure 4E) and *Exploring* (Figure 4F; Supplementary Table 2B). No differences were observed in the remaining behaviors (Supplementary Tables 2A,B).

In summary, the day after delivery in response to a brief separation the EF dams were engaged in behaviors directed to pup care longer than SF. Interestingly, the main differences in maternal behavior between EF and SF dams were found in those behaviors that indicate a more and functional complex maternal care style, as Crouching, Licking and Nest Building.

#### Pups

#### Maternal odor preference T-maze test

**Figure 5:**
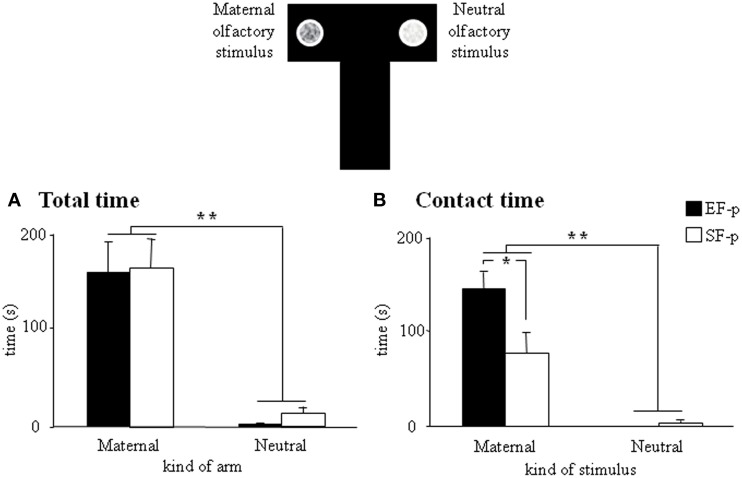
**Maternal odor preference T-maze test**. Results of pre-reproductive maternal rearing condition on pup's discriminative performances are depicted. Histograms show time spent in maternal and neutral arm **(A)**, and time spent in direct contact with the maternal and neutral olfactory stimulus **(B)** analyzed in EF-p and SF-p on pnd 10 (^*^p < 0.05; ^**^p < 0.01). Results are reported as mean ± SEM.

At pnd 10, pups were submitted to a discriminative test of maternal odor. Although all pups spent more *time* in the “maternal” arm (EF-p: Wilcoxon's test, *Z* = 2.80, *p* = 0.005; SF-p: Wilcoxon's test, *Z* = 2.71, *p* = 0.007) and in *direct contact* with the maternal stimulus (EF-p: Wilcoxon's test, *Z* = 2.80, *p* = 0.005; SF-p: Wilcoxon's test, *Z* = 2.59, *p* = 0.009), EF-p contacted the maternal stimulus longer then SF-p did (Mann-Whitney's U, *Z* = 2.11, *p* = 0.03) (Figures 5A,B).

Overall, the test demonstrated the stronger attractiveness of maternal odor for EF-p than for SF-p in the early developmental phases.

#### Sociability test

**Figure 6:**
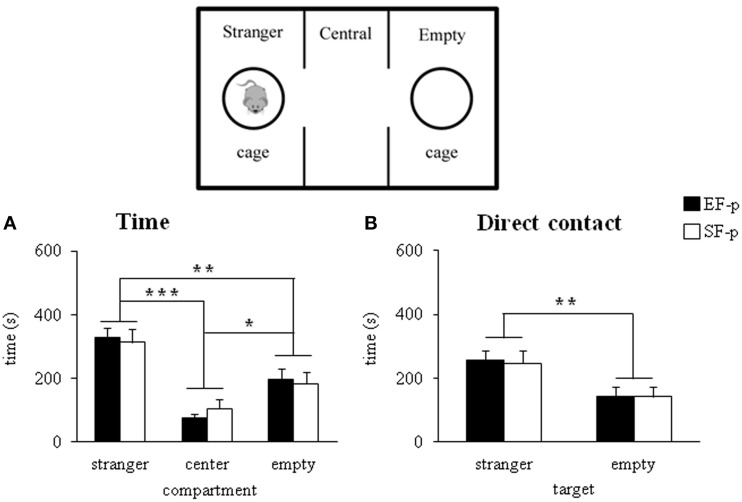
**Sociability test**. Results of pre-reproductive maternal rearing condition on pup's sociability are depicted. Histograms show time spent in the three compartment **(A)**, and in the stranger and empty targets **(B)** analyzed in EF-p and SF-p on pnd 35 (^*^p < 0.05; ^**^p = 0.01; ^***^p < 0.001). Results are reported as mean ± SEM.

During adolescence (pnd 35), both EF-p and SF-p displayed the expected preference for the social stimulus, as revealed by Two-Way ANOVAs (group × compartment) on *total distance* [group: *F*_(1, 22)_ = 0.03, *p* = 0.87; compartment: *F*_(2, 44)_ = 22.22, *p* < 0.000001; interaction: *F*_(2, 44)_ = 0.21, *p* = 0.82] and *time* spent in the compartment with the social stimulus [group: *F*_(1, 22)_ = 1.51, *p* = 0.23; compartment: *F*_(2, 44)_ = 18.92, *p* = 0.00001; interaction: *F*_(2, 44)_ = 0.21, *p* = 0.81] (Figure 6A). Similarly, when the *direct contact* was analyzed, all animals spent more time in the target of the social stimulus [group: *F*_(1, 22)_ = 0.07, *p* = 0.79; target: *F*_(1, 22)_ = 6.84, *p* = 0.01; interaction: *F*_(1, 22)_ = 0.01, *p* = 0.95] rather than the empty one (Figure 6B). No differences between groups were evident also in *frequency of entries* in the lateral compartments [group: *F*_(1, 22)_ = 2.42, *p* = 0.13; compartment: *F*_(1, 22)_ = 1.98, *p* = 0.17; interaction: *F*_(1, 22)_ = 0.19, *p* = 0.66].

In summary, all adolescent pups developed similar sociability performances.

#### Morris water maze

**Figure 7:**
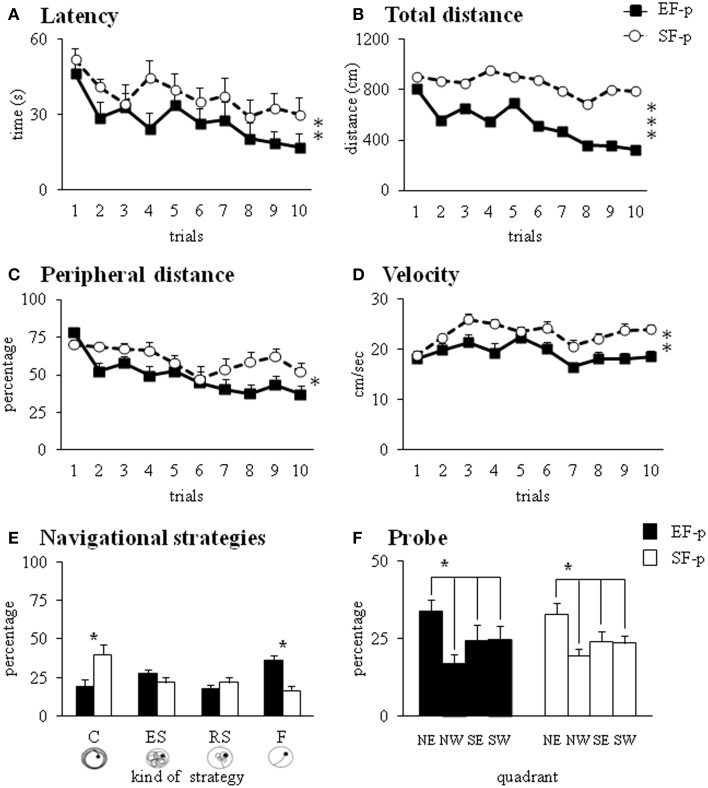
**Morris Water Maze test**. Results of pre-reproductive maternal rearing condition on pup's spatial and procedural performances are depicted. Latency **(A)**, total and peripheral distances **(B,C)**, velocity **(D)**, and navigational strategies put into action **(E)** throughout MWM test. Percentage of time spent in the four quadrants during Probe phase **(F)** analyzed in EF-p and SF-p on pnd 45 (^*^p = 0.05; ^**^p = 0.01; ^***^p < 0.001). Results are reported as mean ± SEM. C, Circling; ES, Extended Searching; RS, Restricted Searching; F, Finding; NE, North-East (previously rewarded) quadrant; NW, North-West quadrant; SE, South-East quadrant; SW, South-West quadrant.

The EF-p employed less time and traveled shorter distances to reach the hidden platform in comparison to the SF-p, as revealed by Two-Way ANOVAs (group × trial) on *latency* [group: *F*_(1, 22)_ = 7.03, *p* = 0.01; trial: *F*_(9, 198)_ = 2.63, *p* = 0.007; interaction: *F*_(9, 198)_ = 0.59, *p* = 0.81] and *total distance* [group: *F*_(1, 22)_ = 13.16, *p* = 0.001; trial: *F*_(9, 198)_ = 1.53, *p* = 0.14; interaction: *F*_(9, 198)_ = 0.60, *p* = 0.79] (Figures 7A,B). Furthermore, Two-Way ANOVAs (group × trial) on *peripheral distance* [group: *F*_(1, 22)_ = 6.18, *p* = 0.02; trial: *F*_(9, 198)_ = 6.13, *p* < 0.000001; interaction: *F*_(9, 198)_ = 1.68, *p* = 0.10] and mean *velocity* [group: *F*_(1, 22)_ = 13.02, *p* = 0.002; trial: *F*_(9, 198)_ = 5.95, *p* < 0.000001; interaction: *F*_(9, 198)_ = 1.62, *p* = 0.11] indicated that EF-p swam less in peripheral sectors and more slowly than SF-p (Figures 7C,D). Significant differences were found in the *navigational strategies* put into action [group: *F*_(1, 22)_ = 1.69, *p* = 0.21; strategy: *F*_(3, 66)_ = 1.88, *p* = 0.14; interaction: *F*_(3, 66)_ = 7.12, *p* = 0.0003], with EF-p showing higher percentages of *Finding* (*p* = 0.03) and lower percentages of *Circling* (*p* = 0.05) than SF-p (Figure 7E). Finally, Two-Way ANOVA on *time* spent in searching the platform during the Probe phase failed to show any significant difference between groups [group: *F*_(1, 22)_ = 0.27, *p* = 0.61; quadrant: *F*_(3, 66)_ = 3.72, *p* = 0.02; interaction: *F*_(3, 66)_ = 0.23, *p* = 0.88] (Figure 7F).

In summary, at pnd 45 EF-p displayed better spatial learning performances than SF-p in the MWM.

#### Open field with objects

In S1, no significant difference between groups was evident on motor parameters [*total distance*: *F*_(1, 22)_ = 1.47, *p* = 0.24; number of *rearing*: *F*_(1, 22)_ = 0.13, *p* = 0.72]. All animals exhibited comparable levels of anxiety, as indicated by the absence of significant differences between groups on emotional parameters [*peripheral distance*: *F*_(1, 22)_ = 1.83, *p* = 0.19; number of *central crossing*: *F*_(1, 22)_ = 0.07, *p* = 0.80; *motionless time*: *F*_(1, 22)_ = 0.01, *p* = 0.91; *number of defecation boluses*: *F*_(1, 22)_ = 2.63, *p* = 0.12].

**Figure 8:**
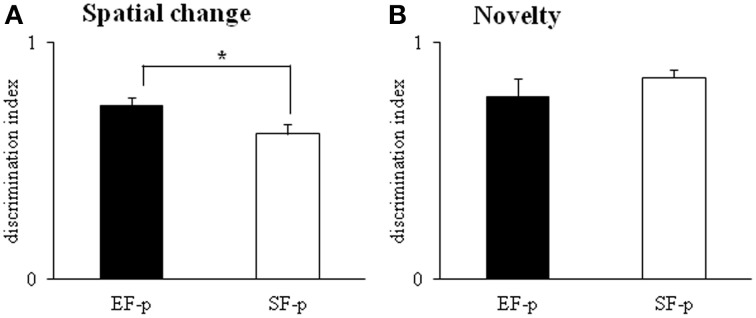
**Open Field with Objects**. Results of pre-reproductive maternal rearing condition on pup's spatial and novelty recognition performances are depicted. Histograms represent discrimination index of spatial change **(A)** and novelty **(B)** analyzed in EF-p and SF-p on pnd 55 (^*^p < 0.05). Results are reported as mean ± SEM.

In S2–S4, all animals showed habituation and progressively decreased object *contact time*, as revealed by a Two-Way ANOVA (group × session) [group: *F*_(1, 22)_ = 2.76, *p* = 0.11; session: *F*_(2, 44)_ = 45.29, *p* < 0.000001; interaction: *F*_(2, 44)_ = 2.51, *p* = 0.09]. In S5 (spatial change), EF-p showed a value of *discrimination index* significantly greater than SF-p [One-Way ANOVA: *F*_(1, 22)_ = 4.53, *p* = 0.04], indicating their marked preference to explore the displaced objects (Figure 8A). In S6, no difference between groups was observed [One-Way ANOVA: *F*_(1, 22)_ = 0.29, *p* = 0.60]. When a new object was inserted into the arena (S7), all rats recognized the novelty and preferred to explore the new object more than the familiar ones [One-Way ANOVA: *F*_(1, 22)_ = 0.94, *p* = 0.34] (Figure 8B).

In summary, at adulthood EF-p showed a more pronounced recognition of spatial rearrangement in comparison to SF-p.

### Biochemical assays

#### Adult neurogenesis

In order to evaluate the effect of pre-reproductive exposure to EE of female rats on the adult neurogenesis of their offspring, the proliferation and differentiation parameters in newly generated neurons of the dentate gyrus were analyzed at pnd 55. No significant differences in the absolute number of proliferating BrdU^+^ newborn neurons were found between EF-p and SF-p groups (*T* = 0.32, *p* = 0.75) (Supplementary Figure 2A). Moreover, no differences were found in the total number of early differentiating progenitors DCX^+^ (*T* = −0.49, *p* = 0.63), of early differentiating newborn neurons BrdU^+^DCX^+^ (*T* = 0.79, *p* = 0.43) (Supplementary Figures 2B–C).

#### BDNF protein levels

#### Dams

**Figure 9:**
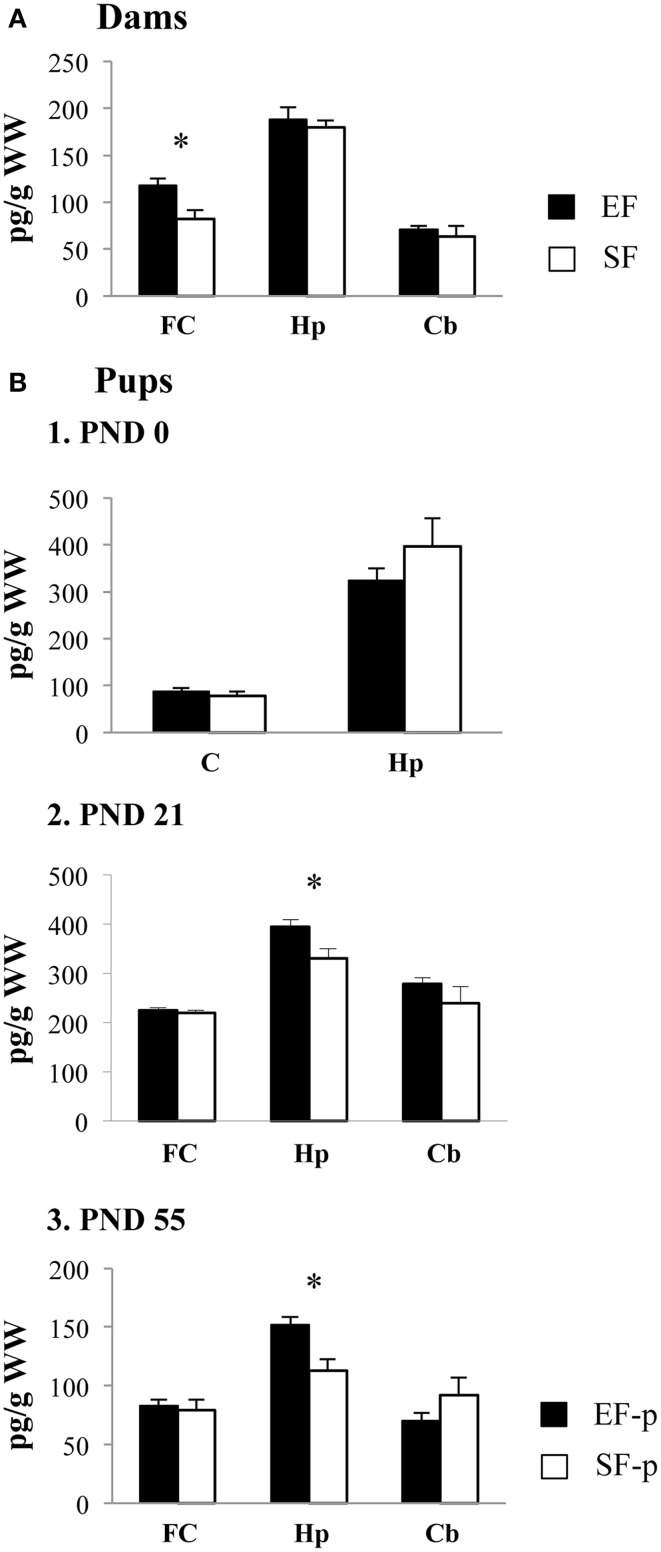
**BDNF protein levels**. Results of pre-reproductive maternal rearing condition on BDNF expression are depicted. Histograms show: BDNF expression levels in frontal cortex, hippocampus and cerebellum of EF and SF at ppd 21 **(A)**; BDNF expression levels in cortex and hippocampus of EF-p and SF-p at birth (pnd 0, **B1**); BDNF expression levels in frontal cortex, hippocampus and cerebellum of EF-p and SF-p at weaning (pnd 21, **B2**) and early adulthood (pnd 55, **B3**) (^*^p = 0.05). Results are reported as mean ± SEM. FC, Frontal Cortex; Hp, Hippocampus; Cb, Cerebellum; C, Cortex.

In frontal cortex EF had significantly higher BDNF levels than SF, as revealed by one-ANOVA [*F*_(1, 6)_ = 8.59, *p* = 0.03], while no significant differences in the BDNF levels were found in hippocampus [*F*_(1, 6)_ = 0.34, *p* = 0.58] and cerebellum [*F*_(1, 6)_ = 0.36, *p* = 0.57] between groups (Figure 9A).

#### Pups

At pnd 0, no significant differences between SF-p and EF-p were found in cortical [*F*_(1, 6)_ = 0.71, *p* = 0.43] and hippocampal [*F*_(1, 6)_ = 1.21, *p* = 0.31] BDNF levels (Figure 9B1).

At pnd 21, EF-p had BDNF levels significantly higher than SF-p in hippocampus [*F*_(1, 6)_ = 6.92, *p* = 0.04]. No significant differences in the BDNF levels were found in frontal cortex [*F*_(1, 6)_ = 0.20, *p* = 0.67] and cerebellum [*F*_(1, 6)_ = 1.18, *p* = 0.32] between groups (Figure 9B2).

Similarly, at pnd 55 EF-p had BDNF levels significantly higher than SF-p in hippocampus [*F*_(1, 6)_ = 10.78, *p* = 0.02], while no significant differences in the BDNF levels were found in frontal cortex [*F*_(1, 6)_ = 0.16, *p* = 0.70] and cerebellum [*F*_(1, 6)_ = 1.89, *p* = 0.22] between groups (Figure 9B3).

#### Levels of reelin protein

No significant differences in reelin levels were found at pnd 0 (hippocampus: *T* = 1.48, *p* = 0.20; cortex: *T* = 1.40, *p* = 0.25) as well as at pnd 55 (hippocampus: *T* = 1.91, *p* = 0.13; frontal cortex: *T* = 1.93, *p* = 0.13) between groups (Supplementary Figures 3A,B).

## Discussion

This study aimed at evaluating whether and how positive pre-reproductive maternal experience influences maternal behavior and offspring phenotype. The pre-reproductive maternal enrichment did influence both maternal care and pups' discriminative and mnesic performances across life. In fact, in comparison to standard females enriched dams showed a more complex maternal care repertoire, as indicated by their higher levels of pup-directed behaviors and nest building activities. The offspring of enriched females showed better discriminative performances in the maternal odor preference T-maze as pups, and higher spatial performances in the MWM at late adolescence and in the OF when adults. Conversely, during adolescence no differences between enriched and standard females' offspring were found in the social abilities.

The superior behavioral performances of enriched females and their offspring were accompanied by profound rearrangement of the neurotrophin pattern. While enriched dams expressed higher BDNF levels in the frontal cortex, their offspring expressed higher BDNF levels in the hippocampus from weaning to adulthood. Conversely, offspring reelin levels and adult neurogenesis were not influenced by the maternal rearing conditions.

### Behavioral performances

In the rat, maternal care influences hippocampal development and thus the functions of spatial learning and memory (Bredy et al., 2004). Namely, the offspring of mothers that exhibit increased levels of pup licking/grooming and arched-back nursing (high LG-ABN mothers) show increased hippocampal N-methyl-d-aspartate (NMDA) subunit mRNA expression, enhanced synaptogenesis and improved hippocampal-dependent spatial learning in comparison to animals reared by low LG-ABN mothers (Francis and Meaney, 1999; Bredy et al., 2004). Moreover, cross-fostering studies provide evidence for the direct effect of maternal care on hippocampal development and cognitive and emotional functions of the offspring (Francis et al., 1999; Liu et al., 2000). Interestingly, in the present study at ppd 1 the enriched females showed increased Licking frequencies and Crouching duration. These behaviors were associated to high levels of nest building activities either at ppd 1 and later at ppd 11–12. Thus, pre-reproductive EE exposure seems to have qualitatively shifted maternal care features to more complex patterns, namely increasing early pup-directed behaviors, similarly to the ones more consistently taken into account in the studies by Meaney's group (i.e., LG and ABN; Caldji et al., 1998), and maintaining high pup care proneness also during later stages of lactation (i.e., nest building).

Overall, these differences in the maternal care may have contributed in changing the developmental trajectories of the pups. In fact, enriched females' offspring resulted more competent in the hippocampal-dependent MWM and OF tests. Interestingly, these better offspring performances were accompanied by increased hippocampal BDNF levels, a well-known modulator of neuronal neuroplasticity, especially in the hippocampus, and thus key regulator for synaptic circuits underlying memory functions (Lu et al., 2005).

Furthermore, in the maternal odor preference T-maze enriched females' offspring were more attracted by dam's olfactory stimuli. In this task performances are mainly related to the early and rapid development of an imprinting process ensuring that the infant forms the caregiver attachment necessary for altricial species survival (Moriceau and Sullivan, 2004). In fact, pups' survival is dependent on learning the maternal odor preference/approach. Besides being attractive for the pups, the maternal odor also organizes their social behavior and ensures that they will nipple attach and receive necessary care and warmth. The unique sensory and mnesic network of maternal odor learning by the infant requires the release of copious amounts of norepinephrine by locus coeruleus into the olfactory bulb until pnd 10 (Moriceau and Sullivan, 2004). This process occurs in the absence of the involvement of brain areas that during infancy are still developing and only partially functional and will become important for learning and memory functions in adulthood, such as the amygdala, hippocampus and frontal cortex (Raineki et al., 2010). The strengthening of the maternal odor learning in enriched females' pups may thus be interpreted as a plastic potentiation of the sensory-mnesic circuit involved in neural processes of attachment, even if we cannot exclude that the differences observed between the two groups of pups could be also due to differences in olfactory perception.

### Biochemical correlates

Interestingly, the two regions exhibiting neurotrophin levels influenced by pre-reproductive maternal rearing conditions were the frontal cortex (in the dams) and hippocampus (in the pups). Such areas are not by chance highly sensitive to parental experiences (Arai et al., 2009; Roth et al., 2009; Mychasiuk et al., 2012) and environmental enrichment (Pham et al., 2002; Gelfo et al., 2011; Chourbaji et al., 2012). Frontal cortex plays an active role in modulating maternal care, so that manipulations, such as cocaine treatment or lesions, may significantly impair maternal goal-directed behavior (Slotnick and Nigrosh, 1975; Ferris et al., 2005; Afonso et al., 2007; Febo and Ferris, 2007; Febo et al., 2010). Also human functional MRI studies evidence that mothers responding to infant sensory cues show metabolic activation in anterior cingulate cortex, insula, orbital and ventromedial frontal regions (Lorberbaum et al., 2002; Leibenluft et al., 2004; Nitschke et al., 2004; Ranote et al., 2004; Noriuchi et al., 2008; Strathearn et al., 2008; Swain, 2008). The increased BDNF levels found in the frontal cortex of the enriched dams can be considered an index of increased neuroplasticity mediated by EE, as we previously demonstrated. In fact, enriched male rats exhibit BDNF and NGF up-regulated frontal levels (Gelfo et al., 2011) as well as increased dendritic branching and spinogenesis in the frontal cortex (Gelfo et al., 2009). In the present study, dams' BDNF levels were measured at pups' weaning (≈6 weeks after the end of EE exposure). Thus, if in the enriched females the frontal neurotrophin levels were stably increased, then these levels might represent a sort of “neuroplastic reserve” (Petrosini et al., 2009) to be spent in the later demanding stages of life, as mothering. On the other hand, the mirroring hypothesis is also plausible: the more complex maternal care of enriched dams increased BDNF levels in the frontal cortex. However, we do not exclude that in the enriched dams the enhanced neuroplasticity induced by the previous EE exposure interacts with the subsequent increase in the maternal activity, resulting thus in higher frontal BDNF levels.

In any case, the enhanced neuroplasticity of the enriched mothers went along with the increased neuroplasticity of their pups. In fact, in the offspring the increase in hippocampal neurotrophin expression contributed in developing their better discriminative and mnesic performances. It has been demonstrated that BDNF regulates multiple aspects of hippocampal development and function (Hall et al., 2000). In fact, elevated hippocampal BDNF levels promote neurite outgrowth and synaptic plasticity through regulation of activity-dependent immediate early genes *c-fos* and early-growth-response-gene 1 and 2 (*Egr-1* and *Egr-2*) (Xu et al., 2000; Zagrebelsky et al., 2005). Furthermore, the induction of long-term potentiation (LTP) increases BDNF transcript levels in the hippocampus (Kovalchuk et al., 2002). Whereas the inhibition of BDNF expression impairs learning and memory functions (Korte et al., 1995; Heldt et al., 2007; Tran et al., 2009), hippocampal BDNF injections improve performances in spatial memory and anxiety tests (Cirulli et al., 2004). Intriguingly, offspring of high LG-ABN dams exhibit enhanced performance in spatial learning and memory tests and increased hippocampal BDNF mRNA levels (Liu et al., 2000). Thus, given the mother represents the primary link between environment and pup, and even subtle variations in maternal care may have a profound impact on offspring development, the pre-reproductive maternal enrichment could have modified the features of the early mothering that probably contributed in turn to build a “neuroplastic reserve” in the pups through the enhanced hippocampal BDNF levels, thus ameliorating their mnesic performances.

While at pnd 0 no difference was found in the cortical and hippocampal BDNF levels, the enriched females' offspring displayed increased hippocampal BDNF levels both at weaning and at adulthood. The lack of neurotrophin modifications at birth does not allow ruling out that eventual latent genetic (germ-line) and epigenetic (intrauterine environment) differences in BDNF signaling required the subsequent interaction with the mother to become manifest in term of BDNF expression. However, once the hippocampal BDNF increase (intrauterine environment, maternal care) due to the pre-reproductive maternal enrichment occurred, it was stable from pnd 21 to pnd 55 and not activity-dependent in relation to behavioral testing. The latter point is particularly important given that in our previous study by Caporali et al. (2014) the accelerated acquisition of complex motor performances exhibited by pre-reproductively enriched dams' pups was associated with increased expression of BDNF and NGF in cerebellum and striatum, with no differences in the hippocampus and frontal cortex that were attributed to the prolonged handling of the pups required by the motor experimental protocol. In that case it was hard to determine whether the neurotrophin differences at cerebellar and striatal level caused the differences in motor behavior or vice versa whether the different motor behavior caused the neurotrophin differences. Notably, a strength point of the present research is that the effects induced by pre-reproductive maternal enrichment on the offspring were not influenced by the manipulations linked to behavioral testing, given that the BDNF increase was found in the not-tested siblings of behaviorally tested pups.

Unexpectedly, the pre-reproductive maternal rearing conditions did not influence reelin levels at birth and adulthood as well as adult neurogenesis. During neurodevelopment the glycoprotein reelin serves as a powerful stop signal for radially migrating principial neurons of the cerebral cortex and hippocampus, is crucial for migration of cortical interneurons, and is necessary in regulating cerebellar Purkinje cell migration (Lakatosova and Ostatnikova, 2012). In adulthood the reelin is expressed by GABAergic interneurons and has an important role as signaling protein in dendritic and axonal growth, neurotransmission and adult neurogenesis (Masiulis et al., 2011; Lakatosova and Ostatnikova, 2012). In particular, being expressed by the basket cells in the hilus of the dentate gyrus, reelin is involved in signaling pathways regulating the generation of new granule cells. It has been demonstrated that reelin alters the cell cycle properties of the transiently amplifying progenitors (probably Type-2 cells) and increases the survival of DCX+ immature neurons (Kuhn et al., 1996; Pujadas et al., 2010). It has been also demonstrated that EE modulates reelin expression (Li et al., 2007; Komitova et al., 2013) and adult hippocampal neurogenesis (Mora et al., 2007; Covic et al., 2010). Since we found no effect on both neurogenesis and reelin levels of the offspring, it is possible to advance that the transgenerational effects of pre-reproductive maternal enrichment critically affect the functional neuroplasticity linked to BDNF rather than the structural features linked to reelin and neurogenesis. Namely, we hypothesize that the BDNF modifications were associated to fine plastic changes regarding mature neuronal structure (such as branching and dendritic spine modifications) and changes in neurotransmission, LTP, and memory consolidation.

### Conclusions

Knowing the mechanisms by which gene-environment interplay is achieved allows clarifying the dynamic nature of gene regulation and biological link between experiences of an organism and individual differences in neurodevelopment and behavior (Franklin and Mansuy, 2010). Multiple mechanisms, such as germ-line and somatic transmission, fetal development, and maternal nurturing, influence the transgenerational inheritance of qualities acquired through parental experience (Weaver et al., 2004; Champagne and Curley, 2009; Ho and Burggren, 2010; Meaney, 2010). In the present study environmental manipulation was applied to mothers and not to fathers, thus transgenerational effects could occur only through maternal germ-line, excluding any paternal influence. The beneficial effects of pre-reproductive EE maternal exposure could have been transmitted to the offspring before the birth *in utero* and after the birth by lactation. In fact, heritable epigenetic changes may have occurred through factors (e.g., hormones, antibodies, antioxidants) transmitted from mother to offspring via placenta or milk (Ho and Burggren, 2010). Also the mother-infant interaction during the early life stages has a remarkable impact on offspring phenotype, as indicated by Meaney's group studies (Weaver et al., 2004; Weaver, 2007; Champagne and Curley, 2009). Only more detailed maternal behavior observations, cross-fostering studies, and epigenetic analyses may determine if the transgenerational inheritance is a product of prenatal factors, post-natal experience or both.

Overall, the present results demonstrating that pre-reproductive maternal environmental enrichment shapes the offspring phenotype indicate that females that experience a particular environment transgenerationally prepare their progeny to cope with that environment (Debiec and Sullivan, 2014). Intriguingly, the transmission of maternal experience could have broad implications for progeny, improving their adaptive competencies and sculpting their behaviors (Petrosini et al., 2009).

## Author contributions

LP, DC, and FA designed the research; DC, PC, DL, and FF performed behavioral evaluation; FG, PD, SF, EB, and FA performed biochemical analyses; DC, PC, FG, SF, EB, MM, and LP analyzed data; all authors discussed data; DC, LP, FG, SV, and EB wrote the paper.

### Conflict of interest statement

The authors declare that the research was conducted in the absence of any commercial or financial relationships that could be construed as a potential conflict of interest.

## Supplementary material

Supplementary Figure 1 **Nest quality photos**. Illustrative photographs representing the different nest quality scores on the basis of the 4-point scale used (0, no nest; 1, flat nest; 3, cup-shaped nest; 4, complex hooded nest).

Supplementary Figure 2 **Adult neurogenesis**. Results of pre-reproductive maternal rearing condition on adult neurogenesis (pnd 55) are depicted. Histograms show: number of proliferating BrdU^+^ newborn neurons **(A)**, number of early differentiating progenitors Dcx^+^ **(B)**, differentiating newborn neurons **(C)**. Representative images show dentate gyrus BrdU^+^DCX^+^ cells of EF-p and SF-p. Scale bar: 25 μm. Results are reported as mean ± SEM. DG: dentate gyrus.

Supplementary Figure 3 **Levels of Reelin protein**. Results of pre-reproductive maternal rearing condition on reelin protein expression are depicted. Representative immunoblots and densitometric graphs of reelin protein levels in hippocampus and cortex at birth (pnd 0) and in hippocampus and frontal cortex at adulthood (pnd 55). Results are reported as mean ± SEM. Hp, Hippocampus; C, Cortex; FC, Frontal Cortex.
