# Supplementary figures and images for: Pre-reproductive maternal enrichment influences rat maternal care and offspring developmental trajectories: behavioral performances and neuroplasticity correlates
Source: Front Behav Neurosci. 2015 Mar 12;9:66. doi: 10.3389/fnbeh.2015.00066 (PMC4357301; doi:10.3389/fnbeh.2015.00066)

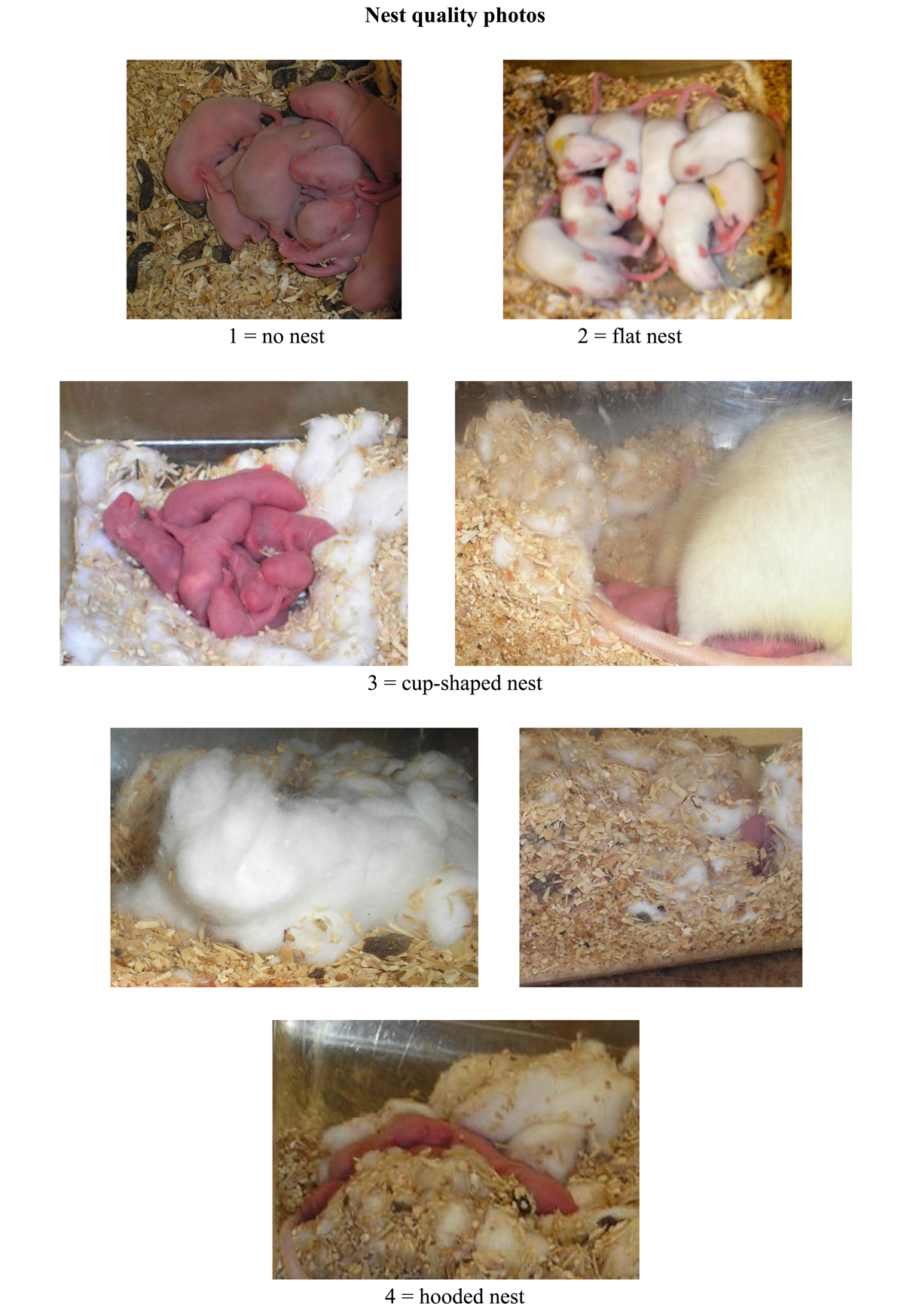

Supplement: Supplementary Figure 1 — Nest quality photos. Illustrative photographs representing the different nest quality scores on the basis of the 4-point scale used (0, no nest; 1, flat nest; 3, cup-shaped nest; 4, complex hooded nest). [file Image1.TIF]

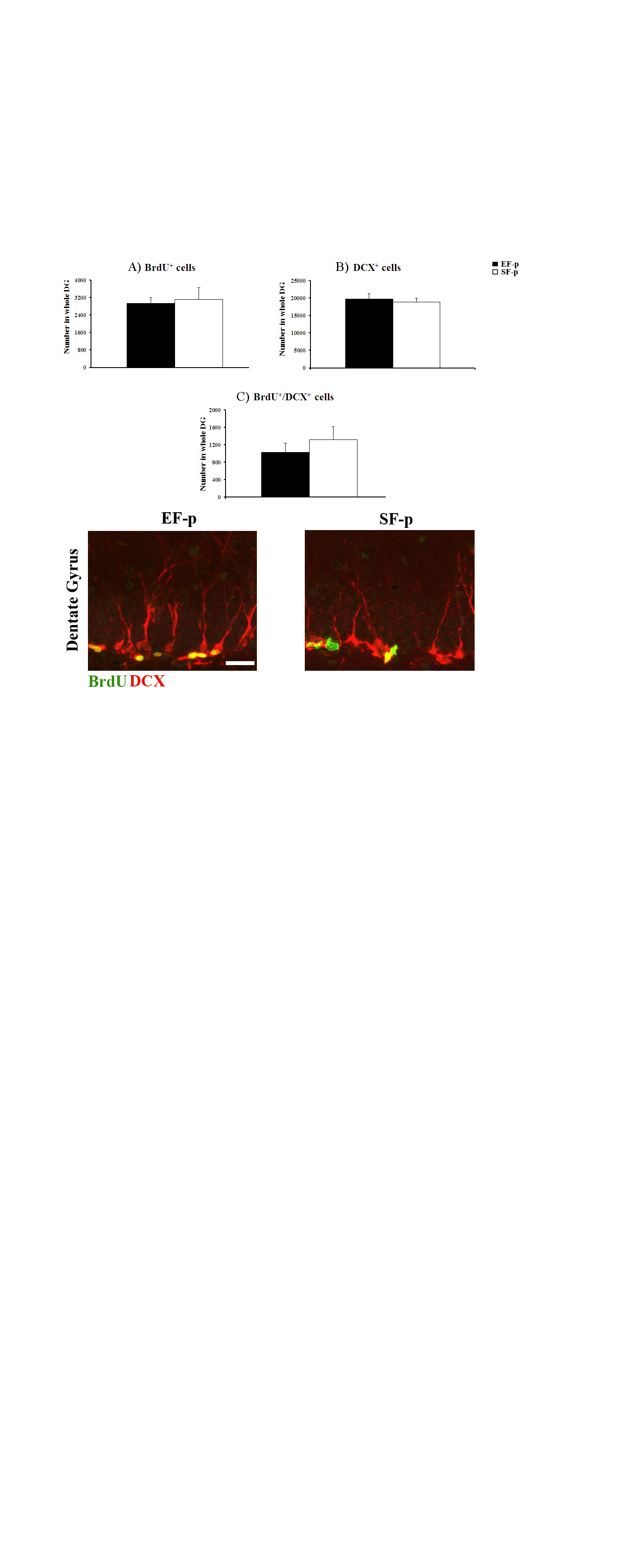

Supplement: Supplementary Figure 2 — Adult neurogenesis. Results of pre-reproductive maternal rearing condition on adult neurogenesis (pnd 55) are depicted. Histograms show: number of proliferating BrdU+ newborn neurons (A), number of early differentiating progenitors Dcx+ (B), differentiating newborn neurons (C). Representative images show dentate gyrus BrdU+DCX+ cells of EF-p and SF-p. Scale bar: 25 μm. Results are reported as mean ± SEM. DG: dentate gyrus. [file Image2.TIF]

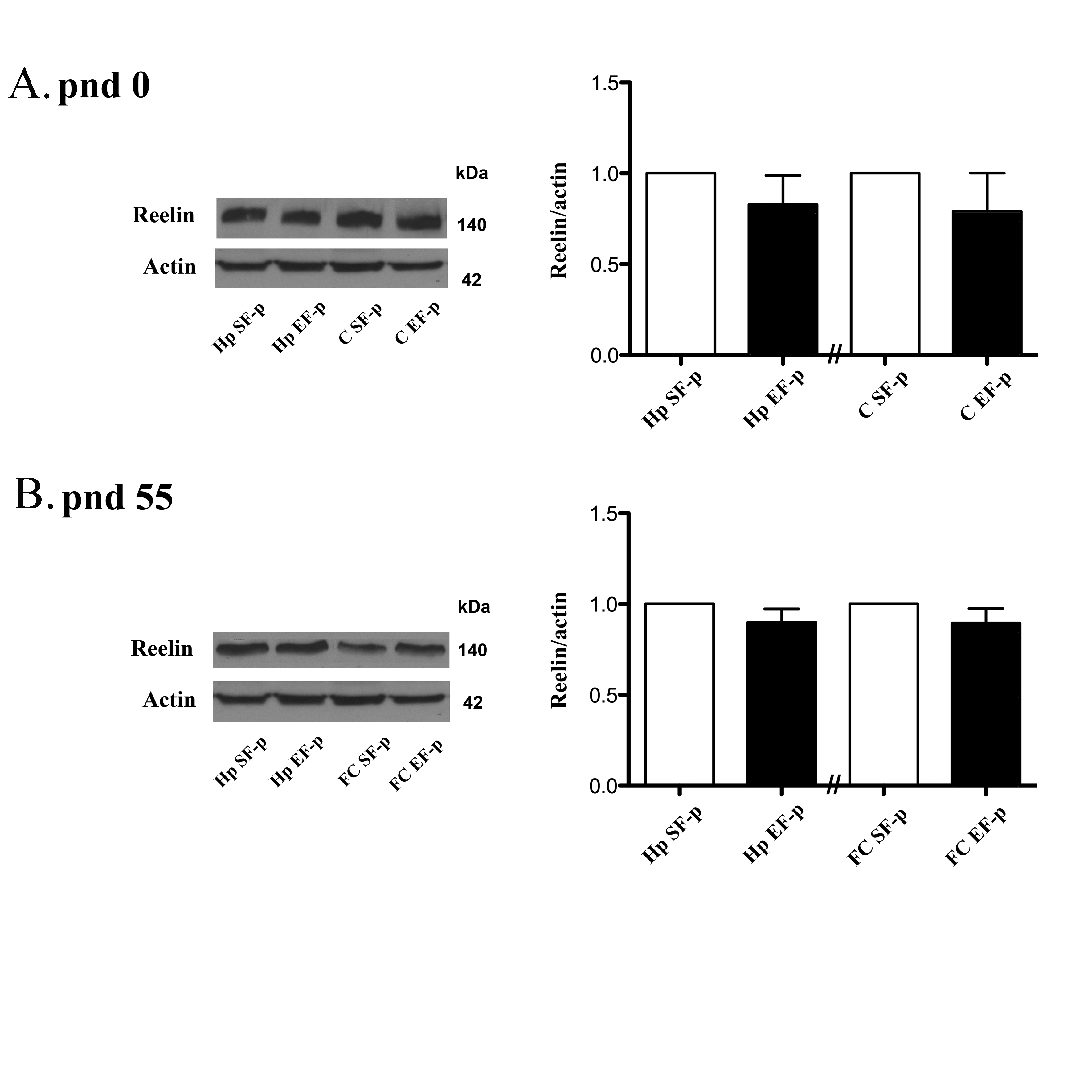

Supplement: Supplementary Figure 3 — Levels of Reelin protein. Results of pre-reproductive maternal rearing condition on reelin protein expression are depicted. Representative immunoblots and densitometric graphs of reelin protein levels in hippocampus and cortex at birth (pnd 0) and in hippocampus and frontal cortex at adulthood (pnd 55). Results are reported as mean ± SEM. Hp, Hippocampus; C, Cortex; FC, Frontal Cortex. [file Image3.TIF]
