# Supplementary material for: Pre-reproductive maternal enrichment influences rat maternal care and offspring developmental trajectories: behavioral performances and neuroplasticity correlates
Source: Front Behav Neurosci. 2015 Mar 12;9:66. doi: 10.3389/fnbeh.2015.00066 (PMC4357301; doi:10.3389/fnbeh.2015.00066)
Supplement: Supplementary file 4 [file Table1.DOCX]

**Supplementary Table 1. Nest building activity.** Statistical significance of EF and SF data comparisons are reported.

| *cotton* *weight* | **ppd1** | **ppd2** | **ppd4** | **ppd6** | **ppd9** | **ppd12** |  | | | | | | |
| --- | --- | --- | --- | --- | --- | --- | --- | --- | --- | --- | --- | --- | --- |
|  | Z=-0.16,  p=0.87 | Z=-1.11,  p=0.26 | Z=-0.86,  p=0.39 | Z=-0.36,  p=0.72 | Z=-1.44,  p=0.15 | Z=-1.56,  p=0.12 |  |  |  |  |  |  |  |
| *latency* | **ppd0** | **ppd1** | **ppd2** | **ppd4** | **ppd6** | **ppd9** | **ppd12** |  | | | | | |
|  | Z=-1.16,  p=0.25 | Z=-1.99,  p=0.06 | Z=-1.25,  p=0.21 | Z= 0.33,  p=0.74 | Z= 0.38,  p=0.70 | Z= 0.51,  p=0.61 | Z=1.00,  p=0.32 |  |  |  |  |  |  |
| *nest quality* | **ppd1** | **ppd2** | **ppd3** | **ppd4** | **ppd5** | **ppd6** | **ppd7** | **ppd8** | **ppd9** | **ppd10** | **ppd11** | **ppd12** | **ppd13** |
|  | Z=-1.15,  p=0.25 | Z=-0.74,  p=0.46 | Z=-0.12,  p=0.90 | Z=-1.48,  p=0.14 | Z=-1.69,  p=0.09 | Z=1.00,  p=0.32 | Z=-0.54,  p=0.59 | Z=-0.87,  p=0.39 | Z=-1.43,  p=0.15 | Z=-1.39,  p=0.16 | Z=-2.16,  **p=0.03** | Z=-2.71,  **p=0.007** | Z=-0.58,  p=0.56 |
| *position* | **ppd1** | **ppd2** | **ppd3** | **ppd4** | **ppd5** | **ppd6** | **ppd7** | **ppd8** | **ppd9** | **ppd10** | **ppd11** | **ppd12** | **ppd13** |
|  | Z=0.64,  p=0.52 | Z=0.00,  p=1.00 | Z=2.04,  p=0.06 | Z=2.04,  p=0.06 | Z=0.64,  p=0.52 | Z=0.64,  p=0.52 | Z=0.00,  p=1.00 | Z=0.00,  p=1.00 | Z=0.00,  p=1.00 | Z=1.68,  p=0.09 | Z=0.00,  p=1.00 | Z=0.00,  p=1.00 | Z=-1.00,  p=0.32 |
| *height* | **ppd1** | **ppd2** | **ppd3** | **ppd4** | **ppd5** | **ppd6** | **ppd7** | **ppd8** | **ppd9** | **ppd10** | **ppd11** | **ppd12** | **ppd13** |
|  | Z=-1.35,  p=0.18 | Z=-1.18,  p=0.23 | Z=-0.53,  p=0.59 | Z=-0.43,  p=0.67 | Z=-1.66,  p=0.10 | Z= 0.00,  p=1.00 | Z=-0.56,  p=0.57 | Z=-0.82,  p=0.41 | Z=-1.05,  p=0.29 | Z=-1.39,  p=0.16 | Z=-1.67,  p=0.09 | Z=-1.28,  p=0.20 | Z=-1.48,  p=0.14 |
| *texture* | **ppd1** | **ppd2** | **ppd3** | **ppd4** | **ppd5** | **ppd6** | **ppd7** | **ppd8** | **ppd9** | **ppd10** | **ppd11** | **ppd12** | **ppd13** |
|  | Z=-0.42,  p=0.67 | Z=-1.26,  p=0.21 | Z= 1.48,  p=0.14 | Z=-1.91,  p=0.06 | Z=-0.53,  p=0.59 | Z= 0.45,  p=0.65 | Z=-0.54,  p=0.59 | Z=-1.44,  p=0.15 | Z=-1.25,  p=0.21 | Z=-0.67,  p=0.50 | Z=-1.73,  p=0.08 | Z=-0.69,  p=0.48 | Z=-0.63,  p=0.53 |
| *additional structures* | **sum (ppd1-ppd13)** |  | | | | | | | | | | | |
|  | Z= 1.80,  p=0.07 |  |  |  |  |  |  |  |  |  |  |  |  |
