# Supplementary material for: Pre-reproductive maternal enrichment influences rat maternal care and offspring developmental trajectories: behavioral performances and neuroplasticity correlates
Source: Front Behav Neurosci. 2015 Mar 12;9:66. doi: 10.3389/fnbeh.2015.00066 (PMC4357301; doi:10.3389/fnbeh.2015.00066)
Supplement: Supplementary file 5 [file Table2.DOCX]

**Supplementary Table 2. Maternal behavior parameters.** Statistical significance of comparisons on EF and SF data. In A, *Pup-directed behaviors*; in B, *Non pup-directed and Other behaviors*.

| **A. pup-directed behaviors** | **1^st^ block** | **2^nd^ block** | **3^rd^ block** |  |
| --- | --- | --- | --- | --- |
| **Sum**  Duration  Frequency  Latency | Z=-0.32, p=0.75  Z= 1.12, p=0.26  Z=-0.48, p=0.63 | Z=-0.32, p=0.75  Z=-0.48, p=0.63  Z=-0.64, p=0.52 | Z=-2.10, **p=0.03** (EF↑)  Z= 2.19, **p=0.02** (EF↓)  Z=-0.49, p=0.62 |  |
| **Retrieving**  Duration  Frequency  Latency | Z= 0.80, p=0.48  Z= 1.04, p=0.31  Z=-0.64, p=0.59 | Z= 1.00, p=0.69  Z= 1.00, p=0.69  Z=-1.00, p=0.69 | Z= 1.00, p=0.69  Z= 1.00, p=0.69  Z=-1.00, p=0.69 |  |
| **Licking**  Duration  Frequency  Latency | Z=-0.80, p=0.42  Z= 0.72, p=0.47  Z= 0.48, p=0.63 | Z=-1.76, p=0.08  Z= 0.00, p=1.00  Z=-0.96, p=0.33 | Z= 1.44, p=0.15  Z= 2.27, **p=0.02** (EF↑)  Z=-1.92, p=0.06 |  |
| **Sniffing**  Duration  Frequency  Latency | Z=-0.32, p=0.75  Z= 0.24, p=0.81  Z=-0.16, p=0.87 | Z=-0.64, p=0.52  Z=-0.80, p=0.42  Z= 1.28, p=0.19 | Z= 0.24, p=0.81  Z= 0.40, p=0.69  Z= 0.40, p=0.69 | |
| **Nursing**  Duration  Frequency  Latency | Z= 1.95, p=0.06  Z= 2.43, **p=0.01**  Z=-1.44, p=0.14 | Z= 0.76, p=0.08  Z= 1.13, p=0.26  Z=-0.80, p=0.42 | Z= 2.56, **p=0.01** (EF↓)  Z= 2.43, **p=0.01** (EF↓)  Z=-1.60, p=0.11 | |
| **Crouching**  Duration  Frequency  Latency | Z=-0.63, p=0.53  Z=-0.63, p=0.53  Z= 0.63, p=0.53 | Z= 0.08, p=0.93  Z=-0.65, p=0.52  Z=-0.08, p=0.94 | Z=-2.24, **p=0.02** (EF↑)  Z=-0.17, p=0.87  Z= 1.60, p=0.11 | |
| **Nest Building**  Duration  Frequency  Latency | Z=-2.24, **p=0.02** (EF↑)  Z=-2.10, **p=0.03** (EF↑)  Z= 0.96, p=0.34 | Z=-1.42, p=0.15  Z=-1.25, p=0.21  Z= 1.43, p=0.15 | Z=-0.81, p=0.42  Z=-0.24, p=0.81  Z= 1.45, p=0.15 | |

| **B. non pup-directed behaviors** | **1^st^ block** | **2^nd^ block** | **3^rd^ block** |  |
| --- | --- | --- | --- | --- |
| **Somma**  Duration  Frequency  Latency | Z= 0.00, p=1.00  Z= 0.48, p=0.69  Z=-1.60, p=0.13 | Z= 0.32, p=0.82  Z= 0.72, p=0.48  Z= 0.48, p=0.69 | Z= 2.42, **p=0.01** (EF↓)  Z= 2.36, **p=0.01** (EF↓)  Z=-1.44, p=0.18 |  |
| **digging**  Duration  Frequency  Latency | Z= 0.32, p=0.75  Z= 0.48, p=0.63  Z=-1.92, p=0.06 | Z=-0.68, p=0.49  Z=-1.12, p=0.26  Z= 1.02, p=0.30 | Z= 1.43, p=0.15  Z= 1.62, p=0.10  Z=-1.92, p=0.06 | |
| **Grooming**  Duration  Frequency  Latency | Z= 0.00, p=1.00  Z=-0.89, p=0.37  Z=-0.80, p=0.42 | Z=-1.76, p=0.08  Z=-0.65, p=0.51  Z= 0.80, p=0.42 | Z= 2.49, **p=0.01** (EF↓)  Z= 2.48, **p=0.01** (EF↓)  Z=-2.48, **p=0.01** (EF↑) | |
| **Wall Rearing**  Duration  Frequency  Latency | Z=-0.32, p=0.75  Z= 1.29, p=0.19  Z=-1.28, p=0.19 | Z= 1.44, p=0.15  Z= 1.22, p=0.22  Z= 0.48, p=0.63 | Z= 1.29, p=0.19  Z= 1.86, p=0.06  Z=-0.32, p=0.75 | |
| **Exploring**  Duration  Frequency  Latency | Z= 1.28, p=0.19  Z= 0.57, p=0.57  Z=-0.80, p=0.42 | Z= 0.16, p=0.87  Z= 0.40, p=0.69  Z=-1.12, p=0.26 | Z= 2.16, **p=0.03** (EF↓)  Z= 2.25, **p=0.02** (EF↓)  Z=-0.99, p=0.32 | |
| **Resting**  Duration  Frequency  Latency | Z=-0.99, p=0.32  Z=-1.09, p=0.28  Z= 1.16, p=0.24 | Z=-0.38, p=0.70  Z=-0.19, p=0.85  Z= 0.00, p=1.00 | Z= 1.48, p=0.14  Z= 1.48, p=0.14  Z=-1.48, p=0.14 | |
| **Other**  Duration  Frequency | Z= 0.72, p=0.47  Z= 1.19, p=0.23 | Z= 0.08, p=0.93  Z= 0.96, p=0.34 | Z=-0.40, p=0.69  Z= 0.64, p=0.52 | |
